# Difficult-to-treat Syndrome of Inappropriate Antidiuretic Hormone Secretion in a Patient with Secondary Central Nervous System Lymphoma

**DOI:** 10.7759/cureus.3905

**Published:** 2019-01-17

**Authors:** Hareesh Joshi, Shonit Nagumantry, Floyd Pierres, Samson O Oyibo, Satyanarayana V Sagi

**Affiliations:** 1 Internal Medicine, Peterborough City Hospital, Peterborough, GBR

**Keywords:** hyponatremia, non-hodgkin's lymphoma, relapse, inappropriate antidiuretic hormone secretion (siadh)

## Abstract

The syndrome of inappropriate antidiuretic hormone secretion (SIADH) is defined as hyponatremia with inappropriately concentrated urine in a euvolemic patient. SIADH is associated with a wide spectrum of clinical conditions. In the hospital, hyponatremia carries significant mortality with a prolonged duration of inpatient stay. It is imperative that the underlying cause is appropriately investigated and such patients are closely monitored. This article presents a case of difficult-to-treat hyponatremia secondary to SIADH in a patient with a rare isolated central nervous system (CNS) relapse from a non-Hodgkin’s lymphoma (NHL). A relapse, particularly affecting the CNS, carries a poor prognosis. The patient was started on dexamethasone and offered treatment with methotrexate but declined. The hyponatremia failed to respond to fluid restriction and demeclocycline. The hyponatremia responded to a single dose of tolvaptan. Clinicians should have a low index of suspicion for a relapse of lymphoma as a cause of difficult to treat hyponatremia in any patient who has previously had remission from lymphoma treatment.

## Introduction

The syndrome of inappropriate antidiuretic hormone secretion (SIADH) is defined as hyponatremia with inappropriately concentrated urine in a euvolemic patient. The reduction of free water clearance is due to increased secretion of the antidiuretic hormone (ADH) from the posterior pituitary despite low plasma osmolality [1]. SIADH is the main cause of hyponatremia in a majority of hospitalised patients [1-2]. The resultant hyponatremia carries significant mortality and is a negative prognostic factor [3].

The clinical presentation depends on the severity and progression rate of the hyponatremia and is dominated by neurological manifestations. Fluid restriction is the recommended initial treatment followed by pharmacologic intervention, including the use of demeclocycline. An alternative treatment option for SIADH is a vasopressin receptor antagonist. It is imperative that careful examination and close monitoring is instituted for such patient groups [3-4].

We present a case with an unusual isolated central nervous system (CNS) relapse of a non-Hodgkin’s lymphoma (NHL) and hyponatremia refractory to fluid restriction and demeclocycline.

## Case presentation

A 76-year-old female presented with episodes of headache and double vision for over one month. Eight months prior to her current presentation, she developed abdominal pain and was found to have a large mediastinal mass with splenic lesions. A biopsy revealed large B cell non-Hodgkin’s lymphoma. Her lactate dehydrogenase at the time of diagnosis was 565 U/L. The patient received six cycles of rituximab, cyclophosphamide, doxorubicin, vincristine, and prednisolone (R-CHOP) followed by radiotherapy to the spleen. Her treatment had concluded two months prior to her current presentation. A whole body fludeoxyglucose positron emission tomography scan showed significant resolution of the mass with no uptake in the spleen. Her past medical history included transitional carcinoma of the bladder for which she had treatment. Examination revealed a reduced level of consciousness with right-sided fifth and sixth cranial nerve palsies.

**Figure 1:**
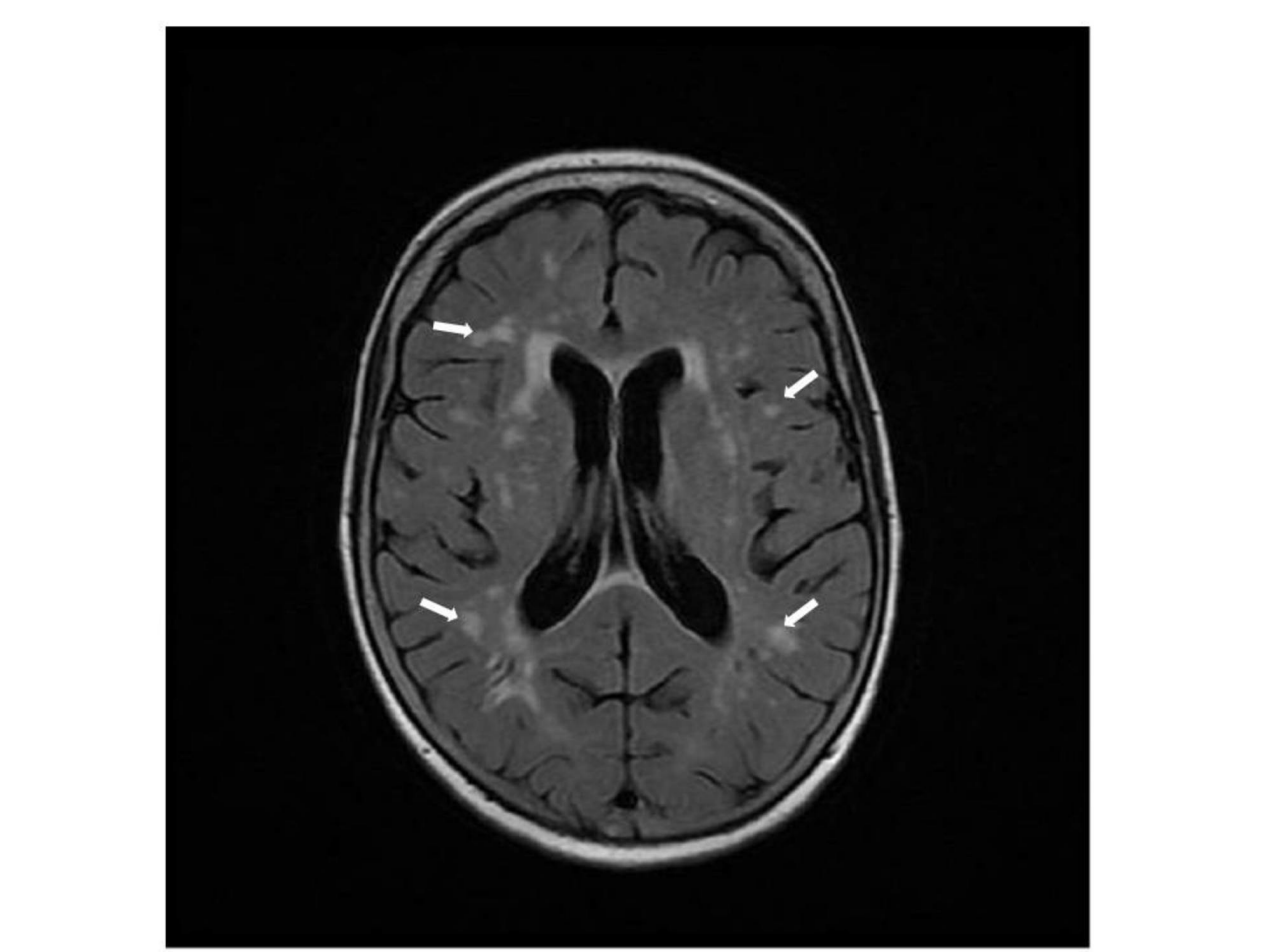
Axial view of magnetic resonance imaging of the brain The image illustrates multiple T2 signal changes (white arrows) consistent with parenchymal infiltration by lymphoma

**Figure 2:**
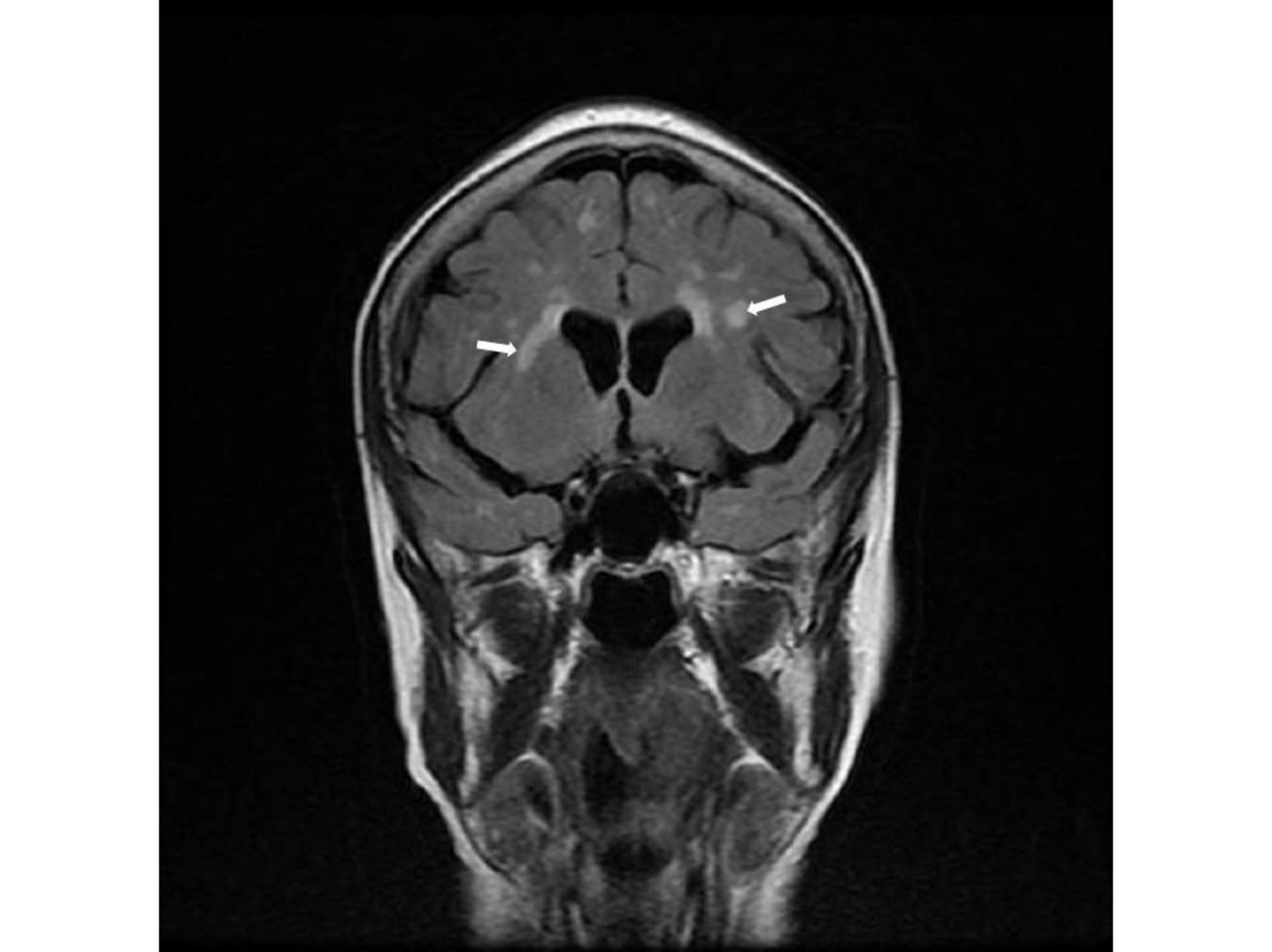
Coronal view of magnetic resonance imaging of the brain The image illustrates similar T2 signal changes (white arrows) consistent with infiltration by lymphoma

Laboratory tests showed severe hyponatremia (serum sodium = 116 mmol/l), low serum osmolality (232 mOsm/kg), inappropriately raised urine osmolality (546 mOsm/kg), and raised urine sodium (54 mmol/L) suggestive of SIADH. A magnetic resonance imaging (MRI) scan of her head revealed abnormal T2 signal changes but no meningeal disease (Figures 1-2). Magnetic resonance angiogram (MRA) of the head and carotids showed no evidence of stroke or dissection. 

**Figure 3:**
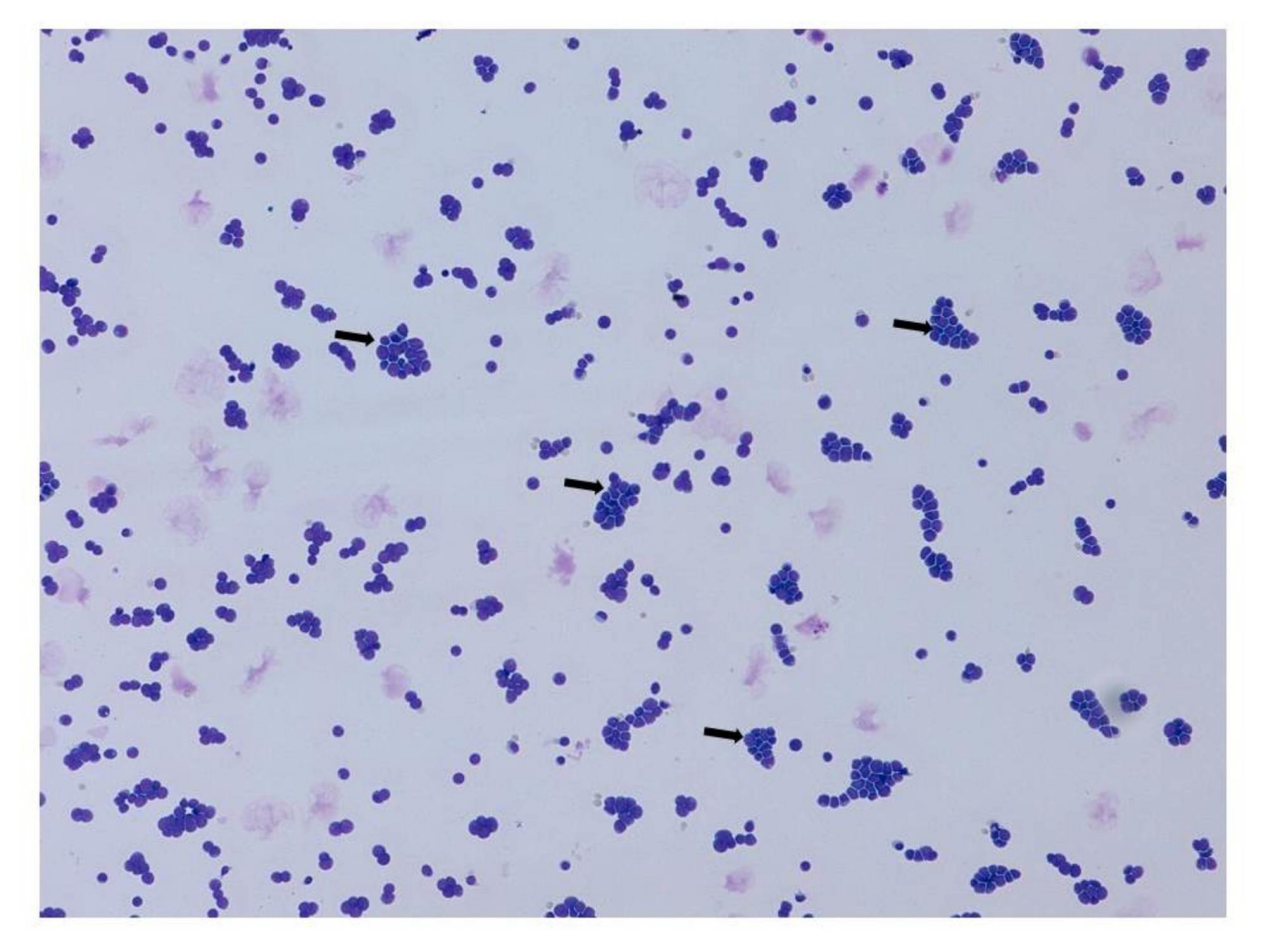
Cerebrospinal fluid cytology The image illustrates abundant lymphoid cells with many larger atypical forms (black arrows) consistent with lymphoma involving the central nervous system

A lumbar puncture for cerebrospinal fluid examination was performed to identify spread from the previously resolved lymphoma and this revealed predominant lymphocytosis with raised protein (2.70 g/L). Further cytological examination revealed atypical lymphoid cells with predominant CD10+ B cells in keeping with the invasion of CNS by lymphoma (Table 1, Figure 3).

**Table 1:** Cerebrospinal Fluid Parameters with Results CNS: central nervous system; RBCs: red blood cells; WBCs: white blood cells

Cerebrospinal Fluid Parameters	Results
Macroscopic Appearance	Clear colourless fluid
Gram Stain	Bacteria not seen
WBCs	544 per microliter
RBC’s	52 per microliter
Polymorphs	5%
Lymphocytes	95%
Culture	No growth after extended incubation
Cellular Pathology	An abundance of lymphoid cells with many larger atypical forms in keeping with lymphoma involving CNS
Flow Cytometry	78% of total events as kappa light chain restricted B cells which express CD10, strong CD38, weak CD43, CD79b, and CD81. These cells do not express CD5, CD20, CD22, CD23, or CD200

**Figure 4:**
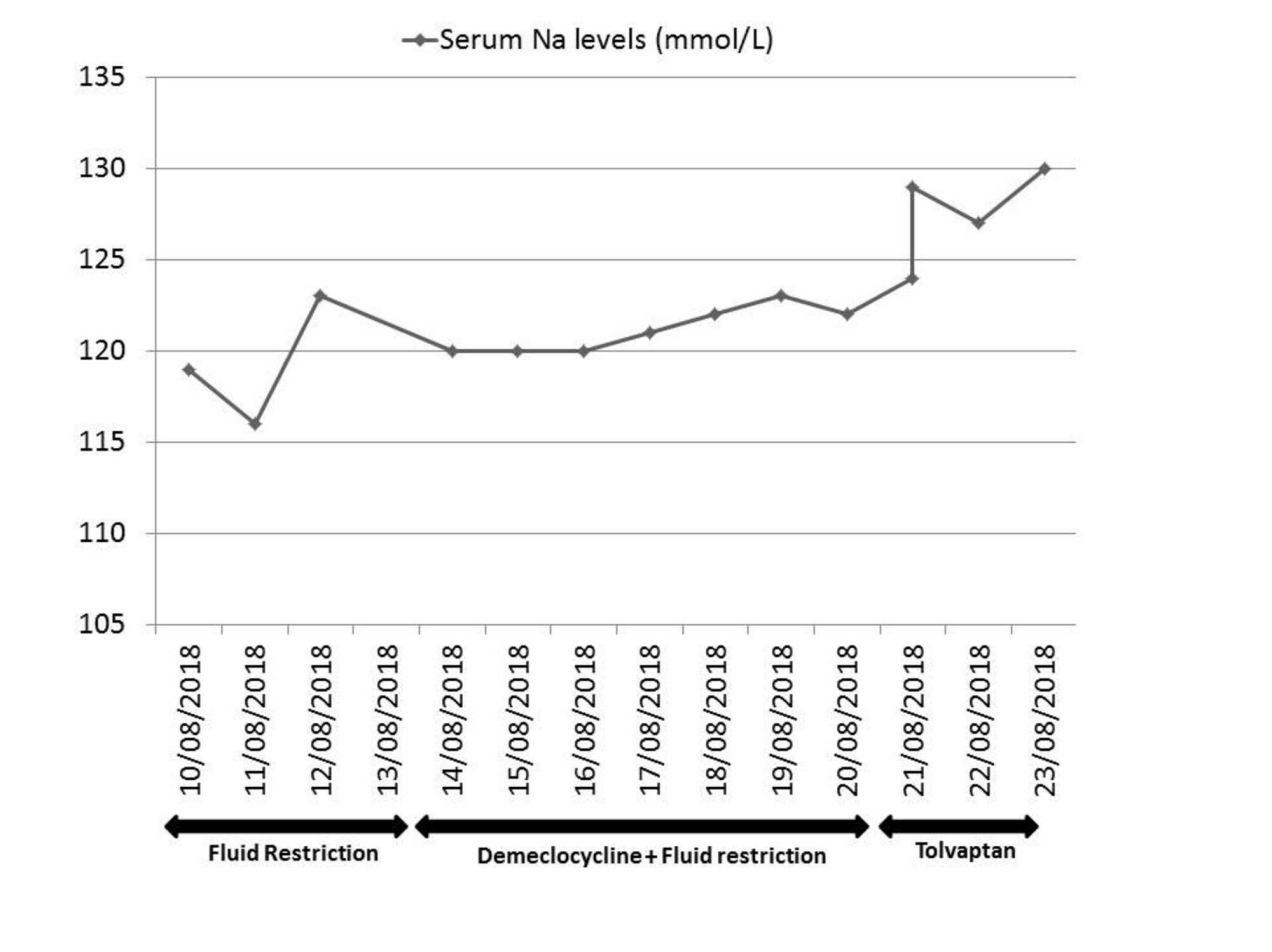
Response to treatment for hyponatremia The graph illustrates initial non-response to first and second line therapy followed by improvement after use of tolvaptan

The patient was managed with dexamethasone, 8 mg twice daily, and fluid restricted to 1 L/24 hrs initially, then to 750 mls/24 hrs for the next four days. On the fifth day, a titrating dose of demeclocycline, 150 mg once daily to 150 mg three times daily, was added with no effect. The hyponatremia responded to a single dose of tolvaptan, 15 mg (increasing to 129 mmol/L within eight hours) and reached normal limits by Day 13 (Figure 4). After a specialist opinion, a palliative approach was taken as the patient had declined further treatment with methotrexate. The patient was kept comfortable in her last days of life and passed away peacefully.

## Discussion

In patients with aggressive non-Hodgkin’s lymphoma, disease relapse of the central nervous system has a poor outcome. An isolated CNS relapse with no evidence of systemic lymphoma at that time is termed as secondary CNS lymphoma. This can involve the brain, parenchyma, leptomeninges, eyes, or spinal cord. It is rare and occurs in less than 1% of patients with NHL. The overall median survival for such patients is four to seven months [5]. Our patient is unusual in that, despite her remission, she had a relapse within two months and within one year of diagnosis. In general, 75% of cases remit to chemotherapy treatment [6]. Furthermore, CNS relapse with meningeal infiltration occurs in only 1% of patients. In addition, this was complicated by hyponatremia likely secondary to SIADH than cerebral salt wasting as the patient was euvolemic with low urine output.

The mechanism of SIADH in our case remains unclear. It is possible that lymphoid cells may have a role in the secretion of ADH or this could be a direct effect of the lymphoid cell invasion of the CNS. SIADH is associated with a wide spectrum of clinical conditions (Table 2).

**Table 2:** Causes of SIADH ADH: antidiuretic hormone; HIV: human immunodeficiency virus; NSAIDs: nonsteroidal anti-inflammatory drugs;  SIADH: syndrome of inappropriate antidiuretic hormone secretion; SSRIs: selective serotonin reuptake inhibitors

Causes	Clinical conditions
Central nervous system disorders	Brain abscess, subarachnoid haemorrhage, encephalitis, Guillain Barre syndrome, head injury, hypothalamic disorders, meningitis, stroke, brain tumour, multiple sclerosis
Lung disorders	Acute respiratory failure, pneumonia, tuberculosis, cystic fibrosis
Malignancies	Brain, lung, stomach, duodenum, pancreas, genitourinary tract, thymoma, lymphomas, sarcomas
Other	Post-surgery, strenuous exercise, malnutrition, HIV infection, general anaesthesia, nausea, pain, stress, mental health disorders, idiopathic
Drugs	Chlorpropamide, SSRIs, tricyclic antidepressants, clofibrate, carbamazepine, vincristine, nicotine, narcotics, antipsychotic drugs, NSAIDs, ADH analogues (desmopressin, oxytocin, vasopressin)

The treatment options for SIADH are usually limited and partially effective. The recommended first-line treatment is fluid restriction. However, as our case demonstrates, this approach had no effect. The tetracycline derivative, demeclocycline, is also used for the treatment of hyponatremia in patients with SIADH. It corrects sodium levels by inducing nephrogenic diabetes insipidus. However, the use of demeclocycline is limited due to the variable onset of action and efficacy. In one systematic review, demeclocycline was found to be effective in only 60% of patients with hyponatremia [7].

Another class of drugs available is the vaptans. These are vasopressin receptor antagonists that compete with ADH at the receptor binding site in the renal collecting duct and cause excretion of free water. Currently, the European Medicines Agency has approved tolvaptan. However, its usage is with caution as the rapid correction of sodium levels can be complicated by osmotic demyelination syndrome (ODS). A case series on adult patients treated with tolvaptan illustrated that the incidence of ODS is higher with the lower start point of sodium levels [8]. In addition, a joint clinical practice guideline published by the European Society of Endocrinology states that tolvaptan is not recommended for the treatment of asymptomatic hyponatremia secondary to SIADH [9]. Our case illustrates that when the first or second line treatment does not achieve clinical results, tolvaptan can be considered. However, adequate rehydration must be ensured before administration to prevent ODS. Our patient may have been slightly dehydrated after a period of fluid restriction, which was stopped prior to commencing tolvaptan. 

Our case is unique for several reasons. First, there was an early relapse after confirmed remission of the lymphoma. Second, this was a secondary CNS lymphoma, which is very rare. Third, the hyponatremia failed to respond to fluid restriction and high-dose demeclocycline. Fourth, the hyponatremia responded gradually to a single dose of tolvaptan.

## Conclusions

This report describes a patient with a rare secondary CNS lymphoma associated with difficult-to-treat hyponatremia. Tolvaptan can be considered where fluid restriction and other treatment modalities have failed with careful monitoring of electrolytes and urine output. Clinicians should consider a relapse of lymphoma as one of the differentials when faced with a patient with difficult-to-treat hyponatremia, even after oncological remission.

## References

[1] Ellison DH, Berl T (2007). The syndrome of inappropriate antidiuresis. N Engl J Med.

[2] Liamis G, Milionis H, Elisaf M (2008). A review of drug-induced hyponatremia. Am J Kidney Dis.

[3] Doshi SM, Shah P, Lei X, Lahoti A, Salahudeen AK (2012). Hyponatremia in hospitalized cancer patients and its impact on clinical outcomes. Am J Kidney Dis.

[4] Fenske W, Störk S, Koschker AC, Blechschmidt A, Lorenz D, Wortmann S, Allolio B (2008). Value of fractional uric acid excretion in differential diagnosis of hyponatremic patients on diuretics. J Clin Endocrinol Metab.

[5] Doolittle ND, Abrey LE, Shenkier TN (2008). Brain parenchyma involvement as isolated central nervous system relapse of systemic non-Hodgkin lymphoma: an International Primary CNS Lymphoma Collaborative Group report. Blood.

[6] Phan J, Mazloom A, Medeiros LJ (2010). Benefit of consolidative radiation therapy in patients with diffuse large B-cell lymphoma treated with R-CHOP chemotherapy. J Clin Oncol.

[7] Miell J, Dhanjal P, Jamookeeah C (2015). Evidence for the use of demeclocycline in the treatment of hyponatremia secondary to SIADH: a systematic review. Int J Clin Pract.

[8] Tzoulis P, Waung JA, Bagkeris E, Carr H, Khoo B, Cohen M, Bouloux PM (2016). Real-life experience of tolvaptan use in the treatment of severe hyponatremia due to syndrome of inappropriate antidiuretic hormone secretion. Clin Endocrinol (Oxf).

[9] Spasovski G, Vanholder R, Allolio B (2014). Clinical practice guideline on diagnosis and treatment of hyponatremia. Eur J Endocrinol.

